# Slitting the Cervix in Dysmenorrhœa

**Published:** 1865-05

**Authors:** 


### SLITTING THE CERVIX IN DYSMENORRHŒA.

CINCINNATI, April 19th, 1865.

E. B. STEVENS, M.D., *Editor Lancet and Observer:*—In a letter received by me a few days since, from Dr. George K. Kidd, Editor of the *Dublin Quarterly*, and one of the physicians to the Coombe Lying-In Hospital, the following passage occurs:—

"You speak of Churchill being opposed to slitting the cervix in cases of dysmenorrhœa. I think I have converted him from this by curing a lady that he had been treating for months, without effect and relief, with sea-tangle. I have no doubt slitting is the only effectual cure in many cases: dilating with tents is utterly useless, as the os closes again in a very few days."

Dr. Kidd's character and position entitle his asertions to be received with great respect. The statement to which Dr. Kidd alludes, was made in one of my letters to the *Lancet & Observer* from Dublin. Immediately after my visit to that city, I spent some little time in Edinburgh, and there had a more favorable opportunity of studying the value of the operation referred to. Professor Simpson, a man not less remarkable for genius than for industry, probably, and justly, the highest living authority in diseases of women, who was the first to perform the operation—now more than twenty years ago—still adheres to it. In what he terms "obstructive" dysmenorrhœa—most authors, however, use "mechanical"—the consequence of narrowing some portion of the cervical canal, generally at the *os externum*, and where, as one of the consequences, there has been sterility, the results of incision have been quite satisfactory. The operation is easily and quickly done with his *hysterotome;* the hemorrhage is but little, unless the incision is very free in the superior portion of the neck, and, even when likely to be severe, can be readily controlled with per-chloride of iron, and, if necessary, the tampon. In one of the cases I saw operated on —the operator was Professor Simpson's nephew, and assistant, Dr. Alexander Simpson—the lady not only walked to the Home, but also walked away, the discomfort and hemorrhage were so slight.

The readers of the *London Lancet* will observe that our own countryman, Dr. Marion Sims, in one of the chapters of his forthcoming book, now in publication in that journal, speaks very positively in favor of the operation.

The treatment of this condition of the cervix by bougies—the advantages gained by sponge-tents or sea-tangle are, as Dr. Kidd so justly observes, but temporary—like most of my professional brethren, I have found very tedious and unsatisfactory. Nor is it, as so well pointed out by Dr. Sims, devoid of danger. It occurred to me, last winter, to see in consultation, a lady with very serious *pelvic cellulitis*, suppuration ensuing, and the pus discharging through the rectum, the disease following the continuous use for several weeks, of bougies, to relieve a contracted cervix. And yet I cannot doubt but in this instance the utmost caution and skill were used with these instruments, for she had been under the care of a leading practitioner in such diseases, in one of our Eastern cities.

In a word, to conclude this note, which was suggested by Dr. Kidd's letter, I am satisfied that the perils of incision are much less than those of dilatation, while the results are far more satisfactory. T. P.

—*Lancet and Observer.*

